# Challenges of immunization in the African Region

**DOI:** 10.11604/pamj.supp.2017.27.3.12127

**Published:** 2017-06-21

**Authors:** Richard Mihigo, Joseph Okeibunor, Blanche Anya, Pascal Mkanda, Felicitas Zawaira

**Affiliations:** 1 WHO Regional Office for Africa, Brazzaville, Congo

**Keywords:** African region, challenges, coverage, equity, immunization, vaccines

## Abstract

Immunization has made significant contribution to public health in the African Region, including elimination, eradication and control of life threatening diseases. Hospitalization due to vaccine preventable diseases has been drastically reduced due to introduction of new effective vaccines. However, optimizing the benefits of immunization by achieving high universal coverage has met with many challenges. The Regional immunization coverage, though raised from its low 57% in 2000 to 76% in 2015 has remained below expected target. Worse still, it has stagnated around 70% for a prolonged period. Cases of inequity in access to immunization service continue to exist in the region. This paper therefore explored the different challenges to immunization in the African Region. Some of the challenges it identifies and discusses include issues of sustainable funding and resources for immunization, vaccine stock-outs, and logistics. Others include data issues and laboratory infrastructure. The paper also attempted some possible solutions.

## Introduction

**Figure 1:**
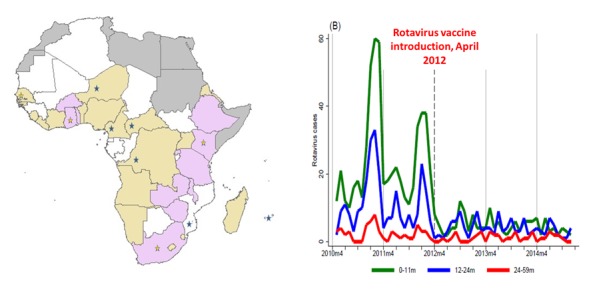
Evidence of new vaccine introduction impact in the African Region

Despite the tremendous progress recorded by immunization programmes, coverage of immunization services has remained suboptimal in the African Region. The past four decades have witnessed advancements in expanding the reach of immunization programmes, and in developing and introducing new vaccines [1]. In the past immunization programmes focused on the infants using mainly a limited number of traditional vaccines. Today, the world of immunization has expanded and there are development and availability of many new vaccines targeting various age groups and populations and also more vaccine preventable diseases [2]. It is hoped that with the introduction of new effective vaccines, such as those against rotavirus and pneumococcal diseases, and enhanced universal and equitable coverage, immunization will considerably contribute to the achievement of the global health goals. Preliminary analysis of 12 countries conducting vaccine impact and effective evaluation as we all 11 countries monitoring intussusception supported by WHO and CDC reveal positive impacts of new vaccine introduction in the African Region. The results show that acute gastroenteritis (AGE) admissions fell by 45%-49% of all admissions among children <5 years following the introduction of Rotavirus vaccine in 2012 in Rwanda (Figure 1). Global guidelines and Regional Strategic Plans were thus put in place to drive the process of reaching high universal and equitable coverage. The Global Vaccine Action Plan (GVAP) 2011-2020 was developed by WHO and UNICEF as a framework from strengthening national immunization programmes towards optimizing the benefits of immunization and achieving a vision of expanded access to vaccines and immunization in an equitable manner [3]. In Africa, the WHO Regional Committee in its 64th session endorsed the Regional Strategic Plan for Immunization (RSPI) 2014-2020, with similar goals and targets as the GVAP [4]. The targets include 90% national coverage and at least 90% in 80% of the districts for vaccines, especially the dose of Diphtheria-Tetanus-Pertussis (DTP3) containing vaccines [3, 4]. The RSPI is employed in developing Country Multi-Year Plans (CMYP) for strengthening national immunization systems to achieve high and equitable immunization coverage in Africa.

Other efforts at boosting the benefits of immunization in the African Region include the facilitation of countries to establish functional National Immunization Technical Advisory Groups (NITAG), to guide policy makers in making evidence based immunization related policy decisions in the context of local epidemiology and cost effectiveness, thus reducing dependency on external bodies for policy guidance. Furthermore, in February 2016, the WHO African and East Mediterranean Regional Offices, in conjunction with the African Union Commission held a Ministerial Conference on Immunization in Africa aimed at sensitizing the political leaders on the benefits of immunization and their role in achieving the global and regional targets [5]. The Region has also intensified collaboration with UNICEF and other partners at promoting community ownership of the immunization programmes to create sustainable demand for immunization services. This is particularly important for increasing demand for and uptake of available services through social and behavioral change interventions; ensuring government transparency and accountability; supporting resource mobilization. Other benefits of involving civil societies and community include influencing national health policies and supporting the monitoring and evaluation of effective programmes. Effective engagement of communities is thus essential to ensuring continued progress toward universal access to immunization. All of these are geared towards achieving high coverage and equity in immunization programmes, considered critical to ensuring immunization for all, in line with the global and regional commitments to protect all against vaccine preventable diseases.

**Figure 2:**
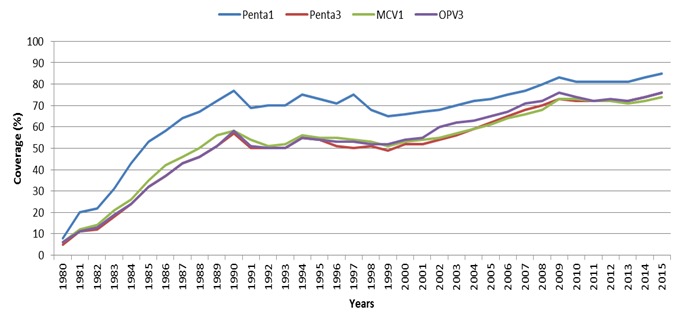
Routine immunization coverage of selected vaccines in the African Region, WUENIC 1980-2015

**Figure 3:**
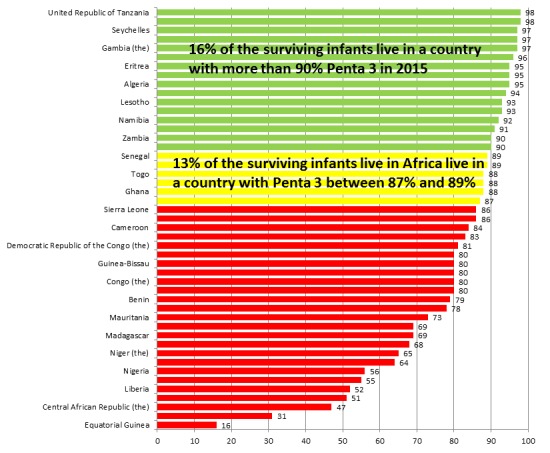
Equity gaps in immunization coverage

Some progress has been recorded in the African Region, following the efforts mentioned above. Immunization coverage has been on a steady rise (Figure 2). For instance, using routine coverage for Diphtheria-Tetanus-Pertussis (DTP), a proxy for immunization performance, immunization coverage has been raised from 57% in 2000 to 76% in 2015 (Table 1, Table 2) [6]. Measles related deaths declined by 86% between 2000 and 2014. Polio is now on the brink of eradication [7-15]. However, in 2014, the number of infants who did not receive the third dose of DTP vaccines in the WHO African Region was estimated to be 7.4 million out of an annual birth cohort of 32.7 million; approximately 23%. Coverage has stagnated at around 70% for a prolonged period [16]. Worse still, there has been significant disparity and inequities in coverage (Figure 3), as coverage is improved in some settings and not in others [16-19]. Today, deaths from vaccine preventable diseases are more where mothers have low education. Children from poorest households are 1.9 times likely to die before age five than their counterparts from richest households; children from rural areas are 1.7 times as likely to die before age five as children from urban areas; under-five years children in fragile contexts are nearly 2 times as likely to die as children of the world [20]. These are reflections of the reach of immunization service and protection of the populations of the African Region with effective vaccines attributable to a number of challenges. This paper gives a brief update on the challenges of attaining high and equitable immunization coverage in the African Region. It will also highlight of possible solutions to the challenges.

**Table 1:** Routine immunization coverage estimates per country in the African Region by all antigens

Country	BCG		Penta 1		Penta 3		MCV1		MCV2		PAB		PCV3		OPV3		RCV1		Rota Last		YFV	
	2015	2014	2015	2014	2015	2014	2015	2014	2015	2014	2015	2014	2015	2014	2015	2014	2015	2014	2015	2014	2015	2014
Algeria	99	99	99	99	95	95	75	68	99	99	92	92			95	95						
Angola	79	81	77	81	64	64	97	97	26		78	78	58	45	70	68			49	18	72	77
Benin	89	93	90	90	79	75	88	88			85	93	74	70	79	75					71	64
Botswana	98	98	98	98	95	95	93	94	85	85	92	92	81	81	96	96			82	82		
Burkina Faso	98	98	95	95	91	91	92	93	50	17	92	89	91	91	91	91	68		91	91	88	88
Burundi	93	92	97	98	94	95	79	80	65	60	85	85	94	95	94	95			96	96		
Cabo Verde	94	99	97	99	93	95	49	49	95	79	92	92			93	95	95	79				
Cameroon	74	82	92	93	84	87	62	54			85	85	85	87	83	86			73	46	77	80
Central African Republic (the)	74	74	69	69	47	47	81	80			60	60	47	47	47	47					48	48
Chad	70	59	60	60	55	46	80	80			75	60			62	54					49	40
Comoros (the)	73	76	81	83	80	80	72	62			85	85			81	79						
Congo (ttie)	85	95	85	95	80	90	79	77			85	85	80	85	80	90			80	60	65	65
Côte d'Ivoire	79	84	99	93	83	76	27	44			85	82	72		81	76					49	49
Democratic Republic of the Congo (the)	74	78	82	81	81	80	85	90			82	82	73	61	78	79					65	65
Equatorial Guinea	48	56	28	59	16	20	78	70			70	70			17	24						
Eritrea	97	97	98	97	95	94	68	61	75		94	94			95	94			96	25		
Ethiopia	75	75	94	86	86	77	97	96			80	80	85	76	85	75			83	63		
Gabon	98	91	87	77	80	70	89	92			85	85			79	68					68	60
Gambia (the)	98	96	99	98	97	96	52	52	77	73	92	92	97	96	96	97			97	92	97	96
Ghana	97	99	97	99	88	98	69	69	63	67	88	88	88	93	88	93	89	92	88	98	88	92
Guinea	72	72	60	60	51	51	75	79			80	80			42	42					53	53
Guinea-Bissau	94	94	92	92	80	80	90	90			80	80	10		78	78					69	53
Kenya	87	94	96	97	89	92	64	58	28		80	76	75	81	83	93			66	19	1	1

**Table 2:** Routine immunization coveraqe estimates per country in the African Reqion by all antiqens (table follows)

Country	BCG		Penta 1		Penta 3		MCV1		MCV2		PAB		PCV3		OPV3		RCV1		Rota Last		YFV	
Lesotho	98	98	98	98	93	93	58	64	82	82	83	83	29		90	90						
Liberia	74	73	77	74	52	50	87	85			89	89	56	45	52	49					56	54
Madaqascar	70	75	79	83	69	73	76	80			78	78	69	72	71	73			69	39		
Malawi	90	97	93	97	88	91	95	95	8		89	89	88	87	88	87			84	83		
Mali	79	79	80	90	68	77	55	60			85	85	58	78	76	84			33	13	64	64
Mauritania	85	98	87	88	73	84	70	84			80	80	71	71	67	84			56	5		
Mauritius	98	97	98	97	97	97	99	98	85	85	95	95			98	98	99	98	66			
Mozambique	95	94	90	92	80	79	85	85			83	83	80	73	80	79			17			
Namibia	94	97	98	92	92	88	85	83			85	85	81		92	88			87			
Niqer (the)	77	76	85	89	65	68	73	72	16	3	81	81	74	13	65	67			70	19	72	70
Niqeria	68	64	70	64	56	49	54	51			55	55	13		55	49					54	51
Rwanda	99	99	99	99	98	98	97	97	87		90	90	98	98	98	98	97		98	98		
Sao Tome and Principe	97	95	98	98	96	95	93	92	76	71	99	99	96	95	96	95					93	92
Seneqal	95	95	94	94	89	89	80	80	54	13	91	91	89	81	85	85	80	80	83		80	80
Seychelles	99	98	99	99	97	99	98	99	98	98	99	99			97	99	98	99				
Sierra Leone	90	90	95	88	86	83	76	78	60		85	85	86	83	86	83			85	53	78	80
South Africa	69	77	72	73	69	70	76	70	63	60	80	80	69	65	70	71			72	72		
South Sudan	43	46	49	49	31	39	20	22			68	64			41	44						
Swaziland	98	99	96	99	90	98	78	86	89	89	88	88	88	67	98	98			36			
Toqo	86	79	92	91	88	87	85	82			81	81	86	34	88	85			85	35	85	82
Uqanda	93	93	89	89	78	78	82	82			85	85	66	50	82	82						
United Republic of Tanzania	99	99	99	99	98	97	99	99	57	29	90	88	95	93	96	97	99	99	98	97		
Zambia	99	99	97	96	90	86	90	85	47	33	85	85	86	77	90	78			82	73		
Zimbabwe	90	99	94	98	87	91	86	92			75	75	87	91	88	92			87	48		

## Methods

**Challenges:** the African Region missed the health-related Millennium Development Goal (MDGs) and has been off-track of the GVAP targets due to some challenges to the immunization programmes. The persistence of these challenges pose a danger to the realization of the Sustainable Development Goals. These challenges include funding shortfalls and a sapping of political will [21]. Others include vaccine stock outs, logistics, etc.

**Logistics:** the introduction of multiple new vaccines has created some challenges for the existing logistics and cold chain requirements due to their current capacity. For instance, the high volume of the prefilled glass syringe presentation of the 7-valent pneumococcal conjugate vaccine outstrips the central cold chain storage capacity of African countries with already weak health systems [22]. Similarly, the safe use and disposal of used glass syringes and needles also pose a waste management challenge [23]. There is need to put in place practical steps for addressing these issues for as many more vaccines get pushed into the system, they become more complex to handle. The systems seem unprepared for the quantum introduction of new vaccines.

**Figure 4:**
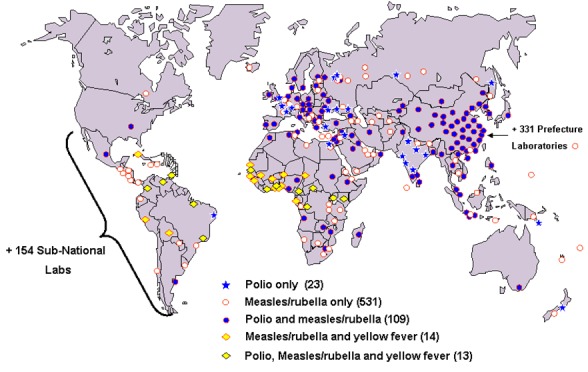
Polio laboratory and surveillance network (>700 labs)

**Laboratories:** much of the laboratory needs for immunization programmes globally and indeed Africa is heavily dependent on the GPEI laboratories for polio eradication. Figure 4 shows that of the over 700 polio laboratory and surveillance network, only 23 are dedicated to polio only. The others service for other vaccine preventable diseases, which are common in the Region. Thus, the polio transition planning and winding down once again brings to fore the weakness of the health systems in supporting immunization activities in the Region.

**Figure 5:**
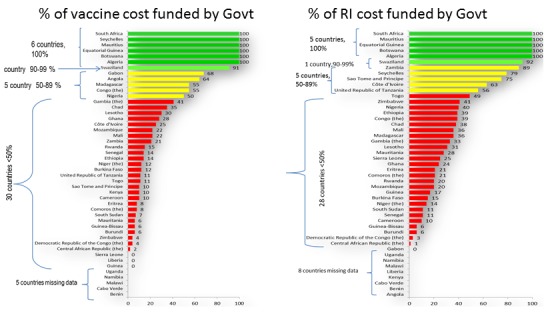
Government funding of vaccine and immunization costs in the African Region 2015

**Funding and commitment**: funding the commitment of national governments for immunization is perhaps one of the most important challenges facing immunization programmes in the African Region today. The cost of vaccinating a child estimated at US$25-$45, baring other costs like training, supervision, communication among others. Several studies suggest that non-vaccine costs represent nearly half of the total cost per child [5, 24-26]. Between year 2016 and 2020, Africa will require a whooping US$17 billion for vaccines and vaccination services [5]. Governments are expected to provide a third of this, which is US$6 billion leaving a gap of about US$5 billion. With increased competition for donor funding for developments and emergencies government commitment and funding of immunization becomes very crucial. Currently, most of the African countries depend of Gavi funds to support vaccine and immunization. Five countries reported funding 100% of their immunization programmes with one funding ≥90%. More than half of the countries (28) are funding <50% of their immunization programme funding needs among which 10 funding even <20% leaving a lot of unmet needs and challenges to reach all target groups [27]. Figure 5 shows a clear picture of the performance of the countries in the African Region with respect to funding immunization. As countries move on the income ladder to the middle income bracket due to improved gross domestic product, and as they graduate out of the Gavi group the availability of resources for immunization will become even more critical. There is thus a dire need for a paradigm shift for governments to begin to take more responsibilities for immunization to ensure sustainable funding. Closely linked to this is the weak involvement of communities in immunization programmes. The role of civil society and community-level work in advancing health, immunization inclusive, cannot be over emphasized. They have their roles in increasing demand for and uptake of available services through social and behavioral change interventions; ensuring government transparency and accountability; supporting resource mobilization; influencing national health policies; and supporting the monitoring and evaluation of effective programmes [5, 28]. Unfortunately the level of involvement of communities and civil societies in immunization services remain weak. Effective engagement of communities is essential to ensuring continued progress toward universal access to immunization. Efforts should be made to explore ways in which community-level efforts, including those by Civil Society Organizations (CSOs), can drive demand for immunization and increase coverage.

Vaccine stock outs: stock-out of vaccine is another major challenge to immunization in the African Region. It has been variously argued that reported stock-out do severe damage to the programmes if the communities are fully mobilized to demand immunization services. Unfortunately, countries continue to report stock-out of vaccines from their health facilities. For instance, in 2015 17 countries in the Region reported at least one episode of stock out of one or more vaccine at national level for duration of at least one week. For some countries, there was also shortage at district level [27]. Further analysis of the data on vaccine stock-out revealed that stock-outs adversely affect vaccination session and constitute disincentive to mothers who bring their children to be immunized.

## Results

### Data as a pillar of immunization coverage

**Figure 6:**
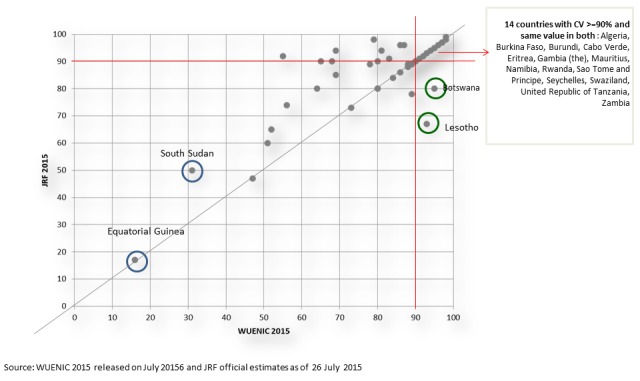
Comparative analysis of data from WUENIC and Administrative reporting

Of crucial importance to building robust national immunization programmes and ensuring the vision of universal health coverage is an increased understanding of national vaccine coverage. Understanding national vaccine coverage is critically important for monitoring the performance of immunization programmes, identifying areas within immunization systems that require improvements, and preparing for the introduction of new vaccines. Consequently, strengthening immunization data quality and coverage estimates, and using the data for improving immunization program performance, are critical steps toward improving coverage in Africa [5]. Participants recognized the role of Ministers, the WHO and private sector in improving access to immunization through strong delivery systems and better data collection, and explored the delivery challenges and opportunities around reaching every child in Africa with the vaccines they need by 2020. Within this context, participants discussed how investments in novel delivery approaches and new data and analytics software are driving local and national progress toward reaching immunization goals. Specifically, the long-term goals for strengthening immunization systems and data collection, their strategies to drive African-led R&D and their visions for African-led solutions as pillars of universal health coverage were discussed exhaustively. Unfortunately, ensuring a system of getting reliable immunization data continue to elude the programmes in the African Region due to many challenges. Some of the challenges include moving denominators and high population movements. Both WHO and UNICEF have continued to work on improving immunization data. WHO/UNICEF estimates of immunization coverage (WUENIC) serves as gold standard against with the administrative reported data are checked for accuracy. A correlation between the WUENIC and the administrative coverage indicates high reliability of the data from the countries. The analysis of DPT3 administrative data (JRF) for 2015 and WUENIC showed similar coverage in 27 countries. The WUENIC for some countries was higher than the administrative data, highlighting possible underreporting from national administrative system. Others seemed to have over reported since the WUENIC was less than the administrative data show (Figure 6).

## Discussion

In this paper some of the challenges to immunization in the African Region were identified and discussed. These include sustainable resources for vaccine and immunization, laboratory facilities for immunization and logistics. Others include the challenges of getting reliable data for planning and coverage as well as persistent vaccine stock-out. The Region has already begun to address some of the challenges. Coming with an insider understanding of the challenges of the burden of communicable diseases [29], needing vaccines, the Regional Director has since taken some bold steps to address the challenges and also sustain the gains of immunization in the African Region. In February 2016, the WHO Regional Offices for Africa and Eastern Mediterranean in conjunction with the African Union and the Government of Ethiopia convoked a ministerial conference on immunization in African towards universal immunization coverage. The conference was an opportunity to bring these challenges to the knowledge of the governments of the African countries and together with political leaders, civil society organizations, community leader think through the challenges and possible solutions. The issue of reliable and sustainable immunization financing, creating demand for immunization, vaccine stock-outs and vaccine production among other issues were exhaustively discussed. At the close of the conference, 49 African countries signed a declaration to address the challenges of immunization in Africa [5]. Furthermore the leadership of the WHO Regional Office has continued to pursue the declaration for full implementation. At the WHO Regional Committee in August 2016, the Regional Director hosted some of the ministers of health to a dinner to discuss the way forward for the ministerial declarations for immunization. In September 2016 partners and a number of member states met to develop roadmap, strategies and discuss monitoring and accountability systems for achieving the demands of the declaration and achieving more fertile condition for the blossoming of the benefits of immunization to all African populations.

With respect to logistic capacities of the countries to WHO is taking a global approach to addressing the issues and countries are being assisted to improve vaccine and waste management and through interaction with industry to seek more suitable formulations and presentations of new vaccines [22]. Similarly, activities are on in the area of surveillance of diseases targeted by new vaccines including enhanced laboratory networks and centres of excellence. When fully developed, these new efforts at enhancing laboratory networks will contribute to alleviating the strain on the health systems following the ramp down of polio resources. In the December 2016 meeting of the Regional Immunization Technical Advisory Group (RITAG) for the African Region, the issue of vaccine stock-out was discussed extensively. The RITAG recognized as a massive disincentive to demand services. It came up with clear recommendations to address it. There were also calls on the Region to begin to consider the situation of vaccine availability especially among Gavi graduating countries. Some African initiatives like AFRIVAC are looked upon to help with cost of vaccines and help mobilize resources for vaccines but there is still need to think of innovative options for vaccine financing. Guaranteed high coverage and equity in immunization programmes is critical to ensuring immunization for all. However, as demonstrated in this paper both equity and high coverage reaching the target levels have been elusive in the immunization programmes of the African Region. Even the current 76% Regional coverage masks national and local inequities and challenges of immunization programmes in the Region [22]. The RITAG recently queried the stagnation of coverage below the desired levels for a prolonged period. It thus called for operational research to know why people are not accepting immunization services as expected.

## Conclusion

For a successful future immunization programme in the Region, there is urgent need to tackle the technical, logistics, political and socio-behavioural impediments to progress in immunization in the Region. As mentioned above, some steps are already being taken to address the challenges at the global, regional and national levels. Globally, the WHO has facilitated consensus, commitment and cooperation among several partners, on vaccines standards, immunization policies and strategic direction in support of countries in this Region as well as other WHO Regions [22]. This support results from the realization that coherent evidence-based policies and well-designed strategic direction are crucial for proving guidance to countries as they make their immunization choices. It remains for the national governments, communities, civil societies and immunization partners to key into the drive of universe access to immunization as a cornerstone for health and development in Africa and each contributing to the project.

### What is known about this topic

- Vaccines and immunization have enhanced the prevention of vaccine preventable disease;
- Progress, in terms increased coverage with traditional vaccines and the newly introduced have been made in immunization programmes in the African Region;
- Coverage has however remained sub-optimal, stagnating around 70% for a prolonged period. Consequently, the targets set for the different vaccines are not met.

### What this study adds

- Challenges of attaining high and equitable immunization coverage, as they relate to the African Region;
- Discuss possible practical steps for addressing the peculiar situation of the African Region.

## Competing interests

The authors declare no competing interest.
